# Utilizing FEM-Software to quantify pre- and post-interventional cardiac reconstruction data based on modelling data sets from surgical ventricular repair therapy (SVRT) and cardiac resynchronisation therapy (CRT)

**DOI:** 10.1186/1475-925X-5-58

**Published:** 2006-10-31

**Authors:** Janko F Verhey, Nadia S Nathan

**Affiliations:** 1 MVIP ImagingProducts GmbH, Nörten-Hardenberg, Germany; 2 Department of Medical Informatics, University Hospital Göttingen, Göttingen, Germany; 3 Department of Anesthesiology, Ohio State University, Columbus, Ohio, USA

## Abstract

**Background:**

Left ventricle (LV) 3D structural data can be easily obtained using standard transesophageal echocardiography (TEE) devices but quantitative pre- and intraoperative volumetry and geometry analysis of the LV is presently not feasible in the cardiac operation room (OR). Finite element method (FEM) modelling is necessary to carry out precise and individual volume analysis and in the future will form the basis for simulation of cardiac interventions.

**Method:**

A Philips/HP Sonos 5500 ultrasound device stores volume data as time-resolved 4D volume data sets. In this prospective study TomTec LV Analysis TEE^© ^Software was used for semi-automatic endocardial border detection, reconstruction, and volume-rendering of the clinical 3D echocardiographic data. With the software FemCoGen^© ^a quantification of partial volumes and surface directions of the LV was carried out for two patients data sets. One patient underwent surgical ventricular repair therapy (SVR) and the other a cardiac resynchronisation therapy (CRT).

**Results:**

For both patients a detailed volume and surface direction analysis is provided. Partial volumes as well as normal directions to the LV surface are pre- and post-interventionally compared.

**Conclusion:**

The operation results for both patients are quantified. The quantification shows treatment details for both interventions (e.g. the elimination of the discontinuities for CRT intervention and the segments treated for SVR intervention). The LV quantification is feasible in the cardiac OR and it gives a detailed and immediate quantitative feedback of the quality of the intervention to the medical.

## Background

The finite element method (FEM) is a powerful numerical method. It has become the prevalent technique used as an effective tool for analyzing all kinds of physical phenomena in structural, solid and fluid mechanics. Moreover it is used for simulating various processes in engineering in the last four decades. The FEM has been frequently used in the field of cardiovascular mechanics (e.g.: [1,2]). A very comprehensive bibliography to the theme of finite element modelling and simulations in cardiovascular mechanics is given by J. Mackerle [3]. Because the heart muscle is a mixture of different kinds of fibre, coronary vessels, blood and interstitial fluids, the complicated system is not easy to be simulated with numerical methods.

But the present paper is not dealing with the simulation of the heart or part of this. This paper uses the step before the analysis of the clinical data. Generally, in order to simulate cardiac mechanics, FEM models are defined. The hypothesis studied in this article is that these models can be efficiently used to carry out detailed volumetric and geometric analysis.

Cardiac FEM models can be generated either from MRI data (e.g.: [4-6]) or from echocardiographic data (e.g.: [1,7]). Intraoperative TEE is currently available in most cardiac surgical operating rooms. In some centers, intraoperative 3D echocardiography is used to evaluate geometry and to plan surgical interventions prior to LV remodeling surgery. However, quantitation of LV geometry is limited to rather imprecise measures such as ejection fraction [8]. Thus, the cardiac surgeon has no sophisticated, immediate, quantitative analysis of the pre- and post-interventional 3D LV geometry. Intraoperative quantitative analysis of the dynamic behavior of the LV might provide optimal information upon which to base precise patient-specific planning of the surgical intervention, as well as to assess the adequacy of the completed surgical repair. Because the LV cannot be realistically described by a symmetric mathematical model, the modern approach consists of using a FEM mesh which approximates LV geometry [9] or whole heart geometry [10].

Up to now few quantitative analysis studies were carried out with data from echocardiographic devices [11]. The present paper actually shows the possibility for immediate quantitative analysis of 4D structural pre- and post-interventional models based on time-resolved 3D echocardiographic data of two clinical cases. One patient underwent a surgical ventricular repair therapy (SVR) and the other a cardiac resynchronisation therapy (CRT).

### Surgical ventricular repair therapy

Surgical ventricular repair or restoration therapy (SVR) is a surgical procedure to treat congestive heart failure caused by myocardial infarction (heart attack) [12]. Following a heart attack, scar or an aneurysm may develop resulting in an enlarged rounded heart. This may lead to congestive heart failure (CHF). The goal of the SVR is to restore the heart to a more normal size and shape, therefore improving function.

### Cardiac resynchronisation therapy

Patients with LV systolic dysfunction and dilatation frequently have ventricular conduction delays. This is usually manifested in the cardiac sinus rhythm as a left bundle branch block, but it often occurs in patients with narrow QRS complex (time it takes for depolarization of the ventricles) also. This type of conduction abnormality is generally associated with delayed depolarisation and contraction of the lateral LV free wall (ventricular dyssynchrony). Ventricular dyssynchrony might contribute to disease progression.

CRT can resynchronise the ventricular activation pattern by acting as an electrical bypass, thus restoring a more coordinated ventricular contraction, by two main mechanisms: pre-exciting the LV lateral wall with atrial-synchronous left or biventricular pacing and shortening or optimising the atrio-ventricular interval. Pre-excitation of the LV lateral wall allows a more coordinated ventricular contraction and decreases in mitral regurgitation by early activation of the papillary muscle. The optimisation of the mitral valve interval abolishes pre-systolic mitral regurgitation and prolongs the diastolic filling time. Relative optimisation of ventricular loading conditions, as provided by CRT, improves myocardial efficiency at no increased oxygen cost and increases the systolic function with little effect on the diastolic function. In addition to the positive hemodynamic effects mentioned above, CRT reduces sympathetic activity [13,14].

## Materials and methods

For this retrospective study data sets of two patients were used. The data sets were acquired as a matter of routine during the intervention. In the first case (named herein PatSVR) the SVR procedure was performed after an heart attack in conjunction with coronary artery bypass grafting to ensure optimal blood supply to the heart. In the second case (named herein PatCRT) the patient had LV systolic dysfunction. PatCRT underwent the CRT procedure to resynchronise the contraction of the LV.

LV images from clinical TEE data sets were obtained in two patients via the midesophageal window using a Philips 5500 ultrasound system. After induction of general anesthesia and airway protection, the esophagus was intubated using an omniplane TEE probe. 3D TEE data sets of the LV structures including mitral annulus and leaflets, chordae tendinae, papillary muscles and ventricular wall were obtained using the automated Philips acquisition protocol at 10° increment. Images were gaited for both beat-to-beat variability and respiratory motion. In order to facilitate acquisition in the shortest possible timeframe we used the same acquisition protocol as described in a previous publication [7].

For LV geometry reconstruction, the TomTec LV-Analysis TEE^© ^software module [15] was employed. This software runs on a standard Dell Inspirion laptop computer with Microsoft Windows™ XP Professional operating system which imports, analyzes, reports and archives the time-resolved 3D-ultrasound data. All images were previously anonymized. The TomTec system automatically detects endocardial borders and produces a 3D shell reconstruction of the LV in which a landmark-setting method is used [7]. With this landmarking procedure, a time-resolved LV geometric analysis with 20 models per heart cycle was obtained for PatCRT. For PatSVR the data sets consist of 24 time steps for the pre-interventional data set and 18 time steps for the post-interventional data set.

**Figure 1:**
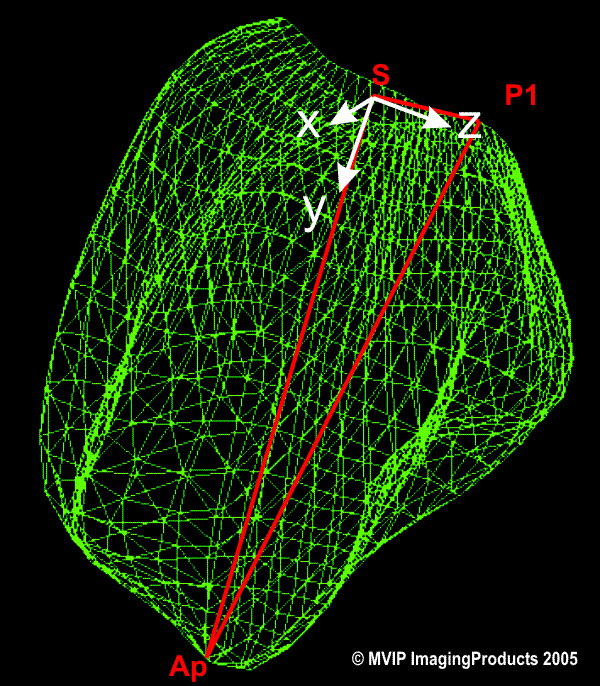
**FEM mesh of the left ventricle**. The triangle defined by Ap (apex), S (center of the mitral valve) and P1 (aortic valve) shows the plane to register. S is the point of origin. The direction from S to Ap gives the direction of the y-axis. The z-axis is in the plane from S in the direction of P1.

**Figure 2:**
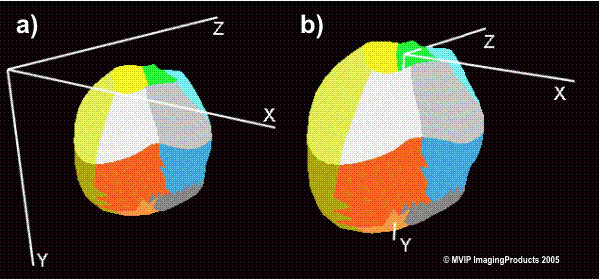
**Registration process**. a) shows the geometry of the LV in the coordinate system prior to the registration. b) shows the same geometry after the registration process. The point of origin is placed in the mitral valve center. In the case the affine transformation matrix consists of a translation component and a very small rotation component. The colored regions show the 16 endocardiac LV segments according to Tomtec LV Analysis.

The rendered LV geometry resulting from the TomTec analysis tool was transferred with FemCoGen 3.0 [16] to an ABAQUS ODB file. In FemCoGen 3.0, the TomTec file structure was reformatted to an ABAQUS system (version 6.5.1) input file based on standard ABAQUS FEM elements. The ABAQUS ODB file was created for visualisation. Furthermore, the LV models were registered according to the positions of the apex, the center of the mitral valve and the position of the aortic valve (Figure 1). The registration process is illustrated in Figure 2. Thus, as a result even the models from two different acquisitions can be overlayed and compared in the same coordinate system.

**Figure 3:**
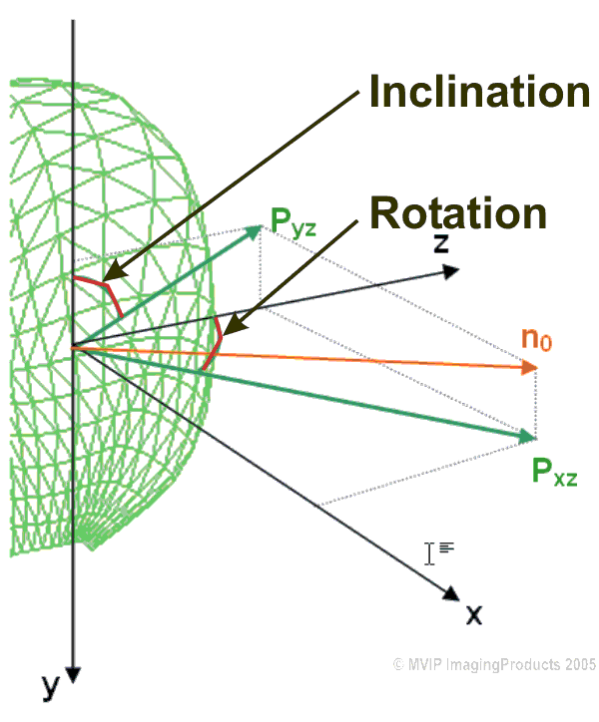
**Rotation angle and inclination angle**. For each segment the average normal vector (n_0_) is calculated. Two projections of n_0 _– P_yz _and P_xz _– are used to calculate the rotation angle (angle between P_xz _and z-axis) and the inclination angle (angle between P_yz _and y-axis).

The transfer was carried out for both, pre- and post-interventional data sets of each patient. FemCoGen 3.0 quantifies partial volumetric and geometric informations. It calculates the partial volumes of each segment as well as two direction angles to quantify the direction of each segment. For the surface of each segment it calculates an average direction numbering the two angles shown in Figure 3. Endocardiac LV pressure was not known for the present data acquisitions. Therefore the length of the vector is normalized to one. Rotation and inclination angles were calculated for comparison purposes which were independent from the length of the normal vector. The quantification results for pre- and post-interventional data obtained with FemCoGen are shown for both patients in the results section.

The LV model consists of 16 segments as previously described [7]. Therein, the apex region consists of four segments (numbers: 13, 14, 15, 16). The wall is described by 12 segments: numbers 2 and 8 for the anterior wall, numbers 3 and 9 for the lateral wall, numbers 4 and 10 for the posterior wall, numbers 5 and 11 for the inferior wall, numbers 6 and 12 for the septum wall and numbers 1 and 7 for the anterior septum wall, respectively. These numbers are used to describe and discuss the findings in the following sections.

## Results

**Figure 4:**
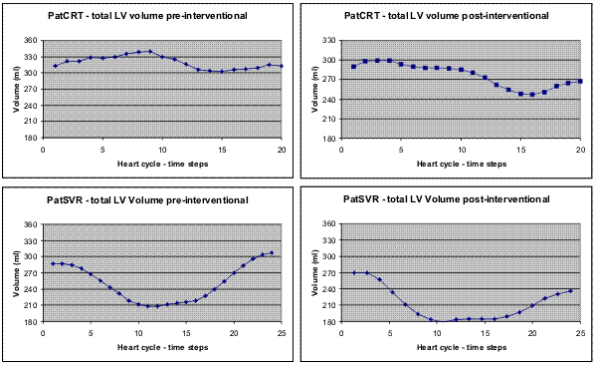
**Total LV volume**. The diagrama show the total LV volume for PatCRT (above) and PatSVR (below). The upper line in each diagram is the total LV volume for the pre-interventional data acquisition, the lower line in each diagram is the total volme for the post-interventional data respectively.

This chapter describes the results for the first quantitative comparison of pre- and post-interventional volumetric and geometric data with FemCoGen 3.0. The total LV volume is shown in Figure 4 for both patients. Generally, the total LV volume decreases after the intervention. For both patients the maximum total volume of the LV at end-diastole (LVEDV) and the minimum at end-systole (LVESV) can be compared. For PatCRT the LVEDV (LVESV) decreases from 339,5 ml (302,3 ml) pre-interventional to 299,6 ml (247,2 ml) post-interventional. For PatSVR it decreases from 307,7 ml (207,7 ml) pre-interventional to 269,8 ml (179,3 ml) post-interventional.

**Figure 5:**
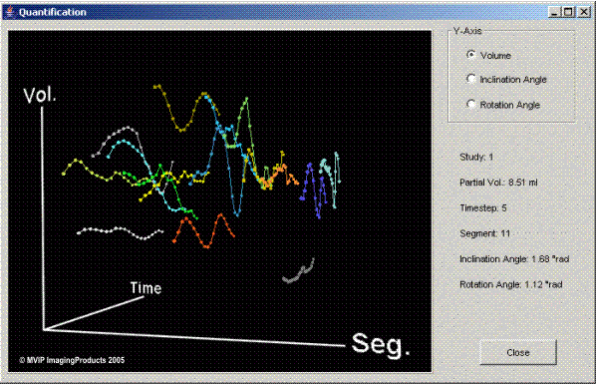
**3D plot of the partial volumes**. This movable 3D plot from FemCoGen shows as an example the partial volumes of the pre-interventional data acquisition from PatCRT. The 16 segments are colour coded and the partial volume for each segment is plotted against the time. Moving the cursor and pointing to each point allows to identify immediately the values for each point in the right part of the frame.

The partial volumes are calculated and plotted in 3D displays (Figure 5). These 3D plots can be manipulated with the mouse and allow direct access to each of the data points. For better presentation in the present paper the display is reduced to 2D plots.

In Table 1 the time steps are correlated with points in the heart cycle.

**Table 1:** Correlation between time steps (frames) and selected points in the cardiac cycle.

	Time step for PatCRT Pre-interventional	Time step for PatCRT Post-interventional	Time step for PatSVR Pre-interventional	Time step for PatSVR Post-interventional
endsystole	18	17	2	2
enddiastole	1	19	1	1
midsystole	7	8	7	6

### Patient PatCRT with cardiac resynchrinsation therapy

**Figure 6:**
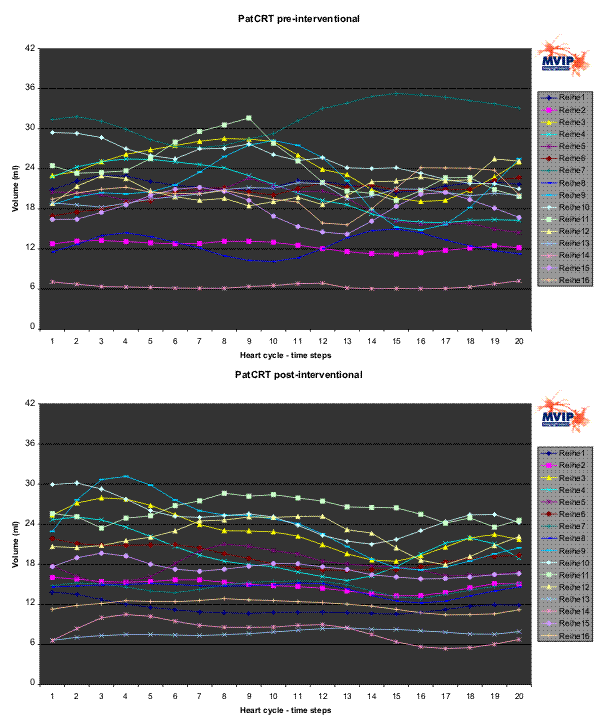
**PatCRT partial LV volumes**. For each segment („Reihe“) and each time step of the cardiac cycle the partial volume is plotted.

**Figure 7:**
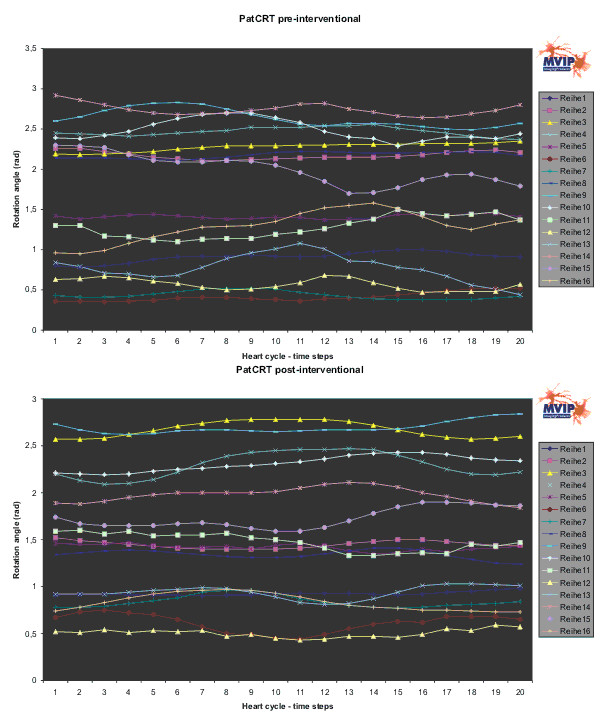
**PatCRT rotation angle**. For each segment („Reihe“) and each time step of the cardiac cycle the rotation angle is plotted.

**Figure 8:**
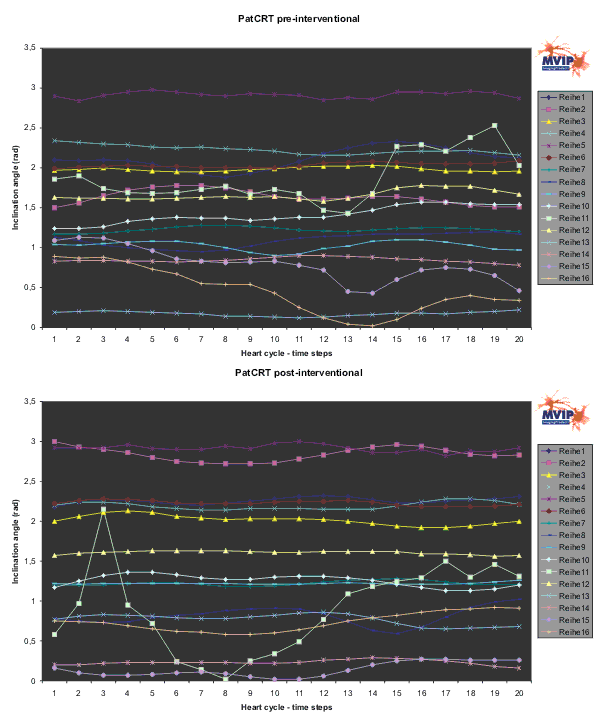
**PatCRT inclination angle**. For each segment („Reihe“) and each time step of the cardiac cycle the inclination angle is plotted.

Figures 6 to 8 show the results for PatCRT. Figure 6 faces the partial volumes of PatCRT for the pre- and post-interventional data acquisition. Figure 7 and Figure 8 show similar results for PatCRT for the rotation angles and for the inclination angles. Some striking changes from pre- to post-interventional partial volume results are observed for (1) volume changing (Figure 6: segments 7, 9 and 11), (2) decrease of the discrete volume changing from time step to time step (Figure 6: segments 10, 11 and 16), and (3) fraction shift of the partial volume (Figure 6: segments 3 and, 9).

Changes in the distribution of the rotation angle (Figure 7) are observed, too. Whereas there is not significant change in the segments around the anterior septum wall 1, 6, 7, 12, and 13, there are some segments with a change of approximately 0.5 rad or 30°. These are some segments on the lateral wall 3, 9, and 14.

For the inclination angle the situation is similar. The segments 1, 2, 6, 7, and 12 around the anterior septum wall show few or no observable changes. Significant changes are observed in the segments 14, 15, and 16, the segments in the apex region. Segment 11 is significantly distorted after the intervention and shows a discrete curvature.

### PatSVR with surgical ventricular repair therapy

**Figure 9:**
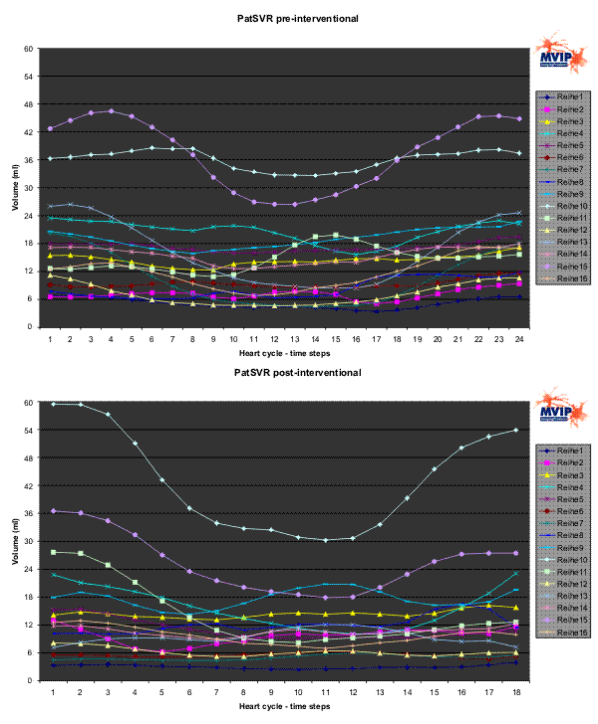
**PatSVR partial LV volumes**. For each segment („Reihe“) and each time step of the cardiac cycle the partial volume is plotted.

**Figure 10:**
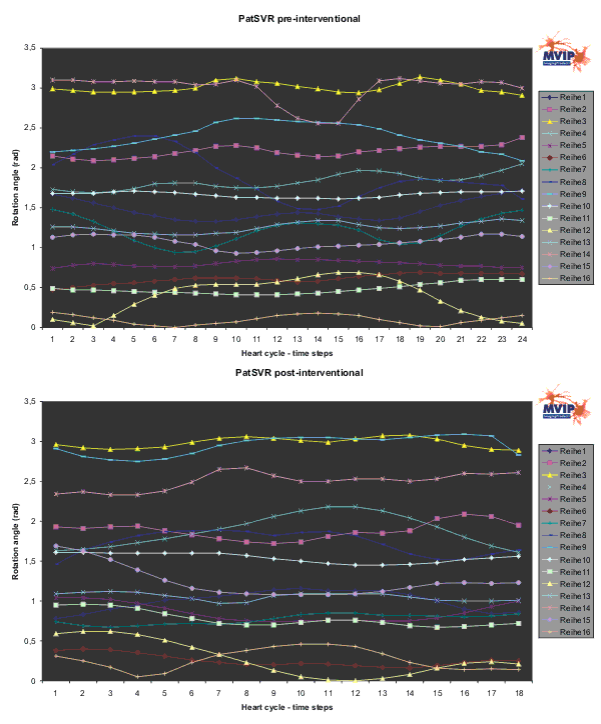
**PatSVR rotation angle**. For each segment („Reihe“) and each time step of the cardiac cycle the rotation angle is plotted.

**Figure 11:**
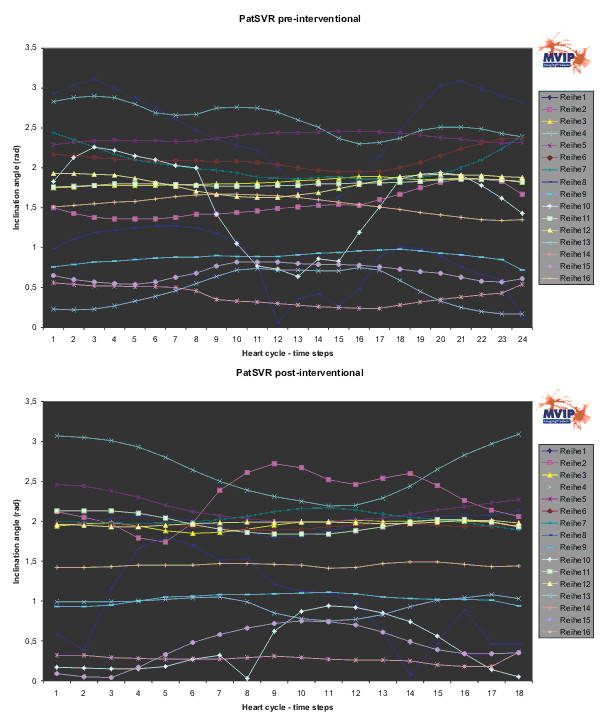
**PatSVR inclination angle**. For each segment („Reihe“) and each time step of the cardiac cycle the inclination angle is plotted.

Figures 9 to 11 show the results for PatSVR. For the partial volumes (Figure 9) most segments have homogeneous curves in the range from 3 ml to 21 ml fraction contribution to the total volume. The segments 10 and 15 behave aberrant from this. The partial volume of these segments is significantly higher than the partial volume of all other segments. After the intervention the partial volume of segment 15 shows a decrease of 6 ml on average. The overall maximal partial volume of segment 10 in the pre-interventional data is ~60 ml, with an average of ~42 ml. This is reduced and balanced after the intervention to an average of 36 ml.

The rotation angle distribution in Figure 10 shows a change of the variance in most segments. Especially, for the segments 8 and 14 this means a reduction of the variance of ~0.25 rad or ~15°. Segment 15 is the only one behaving antidromic with a variance increase almost in the same range. A reduction of the rotation angle on average of approximately 0.30 rad or 18° is observed in the segments 6, 7, 12, and 14. A major decrease of the rotation angle is observed only in the segment 9.

The first qualitative impression of the two diagrams in Figure 11 is of complete disorder due to the high variance of some curves (segments 1, 2, 8, 10), three of them belonging to the anterior/anterior septum wall section. Before the SVR therapy the segments 1, 8 and 10 show a high variance, but after the treatment the high variance changed to the segment 2 and 10. Apart from the segments with a high variance, there are two groups of segments. The first group consists of the segments 3, 5, 6, 7, 11, 12, and 16, with most belonging to the septum and inferior wall section. After the treatment this group shows an average inclination angle of 2 rad and the variance of the entire group decreases from 0,15 rad to 0,11 rad. The second group consists of the segments 9, 13, 14, and 15, most of them belonging to the apex region. After the treatment this group shows an average inclination angle of 0.50 rad and the variance of the entire group slightly increases from 0,11 rad to 0,13 rad.

**Figure 12:**
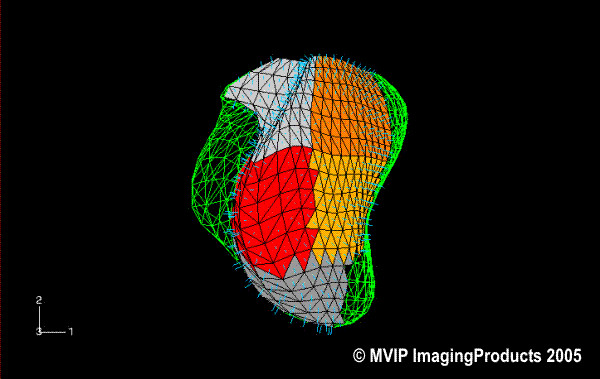
**Visual comparision with ABAQUS**. The finite element modelling tool ABAQUS visualizes pre- and postinterventional data sets. Here the mid-systolic LV models from PatSVR are shown. The wireframe shape shows the LV pre-interventionally and the coloured shape shows the post-interventional LV. The display of the post-interventional shape is limited to the segments treated during the intervention 3,4,9,10,14.

The last figure (Figure 12), shows the qualitative visualisation of pre- and post-interventional data for PatSVR using the finite element visualisation and modelling tool ABAQUS.

## Discussion

For both patients the method described quantifies the operation results post- and pre-interventional: PatCRT data show the elimination of the discontinuities of the partial volumens after the intervention; PatSVR data show the segments treated. They are clearly identified and quantified.

Quantitative pre- and intraoperative volumetry and geometry analysis of the LV is feasible in the cardiac operation room using the technique described. The entire calculation process with FemCoGen takes less than 3 minutes for each data set. To the medical therefore it gives a detailed and immediate quantitative feedback of the quality of the intervention.

To ensure optimal medical treatment, medicine must meet industry-established benchmarks; this is a goal that can be attained only through the ongoing use of appropriate quality management (QM) methods. The technique described in this paper is one step in this direction. It can be part of a QM process that closely resembles those found in industrial settings, where statistical process controls are used to improve quality.

## Authors' contributions

JFV did the technical part analysing the FEM models. NSN acquired the data and did the medical part. Both authors read and approved the final manuscript.
